# Medicolegal autopsy analysis during Covid-19 pandemic in India

**DOI:** 10.6026/973206300212400

**Published:** 2025-08-31

**Authors:** Lairikyengbam Meetali Roy, Rajeshwar Pate, Biyabani Naushad Husain, Mangesh Ghadge, Aswin Kennedy

**Affiliations:** 1 Deparment of Forensic Medicine, KEM Hospital, Mumbai, India; 2 Deparment of Forensic Medicine, Rajiv Gandhi Medical College, Thane, Maharashtra, India; 3 Deparment of Forensic Medicine, Hi-Tech Medical College, Rourkela, Odisha, India

**Keywords:** Forensic pathology, forensic medicine, covid-19

## Abstract

Medicolegal autopsy analysis is an integral part of a forensic investigation. Therefore, it is of interest to document the impact of COVID-19 on autopsy analysis. Hence, a total of 1,569 medicolegal autopsies were analysed. Data shows only 6.37% autopsy were conducted during lockdown while it is 93.63% in the unlock phase, indicating significant disruption in forensic services. The pandemic significantly altered the pattern and volume of medicolegal autopsies, with reduced cases during lockdown and a surge post-lockdown, particularly from respiratory, cardiac, suicidal and trauma-related deaths. Shifts in age distribution and operational delays highlight the broader impact on forensic services, underscoring the need for adaptable protocols and integrated public health responses during emergencies.

## Background:

The World Health Organization on 11 March 2020 had declared the severe acute respiratory syndrome coronavirus 2 (SARS-CoV-2) infections as global pandemic [1]. The importance of autopsies is accentuated in metropolitan cities, where the burden of unnatural deaths due to road traffic accidents (RTAs), suicides, homicides and accidental deaths is significantly higher due to urban stressors, high population density and complex sociocultural dynamics [2]. The onset of the COVID-19 pandemic in early 2020 posed unprecedented challenges to healthcare systems globally and forensic services were no exception. The conduct of medicolegal autopsies during the pandemic had to be modified in accordance with new biosafety guidelines to prevent occupational exposure to SARS-CoV-2, especially in suspected or confirmed COVID-19 deaths [3]. The World Health Organization (WHO), the Indian Council of Medical Research (ICMR) and national health agencies issued protocols that limited the extent and number of autopsies, emphasizing external examinations and virtual modalities whenever feasible [4, 5]. In another study, Subsyndromal mental health problems are a common response to the COVID-19 pandemic [6]. The National Crime Records Bureau (NCRB) reported significant increases in suicide rates and accidental deaths during the pandemic, attributed to isolation, financial hardship and limited access to mental health care [7]. Simultaneously, a sharp decline in road traffic accidents was reported during lockdown periods due to restricted vehicular movement, although this trend began reversing as restrictions were lifted [8]. Therefore, it is of interest to document the indirect impact of the pandemic on mortality patterns and the functioning of forensic services.

## Materials and Methods:

The present study is retrospective observational study of medico-legal autopsies performed at the mortuary of Rajiv Gandhi Medical College, Thane during the Covid 19 Pandemic period of March 2020 to March 2022. The total numbers of autopsies conducted were 1569. The postmortem examination was strictly done after going through Covid 19 Antigen test negative report. Information related to autopsies including medical history, place of death, cause of death and manner of death were obtained from completed post mortem reports, inquest reports prepared by inquirer into sudden death and police investigations reports. Anonymity was maintained at all times, denoting each case by a serial number. The nationwide lockdown in India due to the COVID-19 pandemic was implemented in multiple phases during the year 2020. The first phase, Lockdown 1.0, lasted for 21 days from 25th March to 14th April. This was followed by Lockdown 2.0 for 18 days (15th April to 3rd May), Lockdown 3.0 for 13 days (4th May to 17th May) and Lockdown 4.0 for another 13 days (18th May to 31st May). From June 2020 onwards, the country entered the unlock phases, beginning with Unlock 1.0 (1st to 30th June) and Unlock 2.0 (1st to 30th July), each lasting for 30 days. Subsequently, unlock 3.0 commenced from August 2020 and extended through to March 2022, during which restrictions were gradually lifted and activities resumed in a phased manner.

## Results:

A total of 1,569 medicolegal autopsies were conducted during the COVID-19 pandemic at a tertiary care hospital in a metropolitan Indian city. The distribution across the lockdown and unlock phases was markedly uneven, with only 6.37% (n=100) performed during the lockdown, compared to 93.63% (n=1,469) during the unlock phase, highlighting the pandemic's disruption of forensic services (Table 1 - see PDF). Age-wise, autopsies were most frequent among individuals aged 31-40 years (21.10%), followed by 21-30 years (19.38%). Autopsies in younger children (0-10 years) and the elderly (71+ years) were significantly lower. A Chi-square test showed a statistically significant association between age group and pandemic phase (χ^2^ = 17.70, df = 8, p = 0.0236), indicating the age distribution of deaths varied meaningfully between lockdown and unlock periods (Table 1 - see PDF). This shift likely reflects differing exposure risks, mobility patterns and healthcare access during the pandemic. Gender distribution remained consistent across both phases, with males constituting 70.87% of cases and females 29.13%. The Chi-square test did not reveal a significant association between gender and lockdown status (χ^2^ = 0.589, df = 1, p = 0.443), suggesting stable gender trends throughout the pandemic, though the predominance of male autopsies could be due to gendered differences in risky behaviour and occupational exposure (Table 2 - see PDF). Cause of death varied significantly by phase, as reflected in the Chi-square test (χ^2^ = 21.46, p = 0.0181). Respiratory-related deaths (tuberculosis and pneumonia) rose sharply from 1.08% during lockdown to 11.22% in the unlock phase, totalling 12.30%. Cardiac causes (*e.g.*, coronary artery disease) followed a similar trend, increasing from 1.34% to 14.02%, accounting for 15.37% of total autopsies. Deaths from hanging also rose sharply, comprising 11.85% overall, with a disproportionate increase during the unlock phase (11.15% vs. 0.70%).

Trauma-related causes-head injury with polytrauma (9.05%) and haemorrhagic shock (7.84%)-increased significantly during unlock likely due to resumed traffic and social activity (Table 3 - see PDF). A large proportion of cases (34.42%) were categorized under "opinion reserved/pending," the majority (32.32%) from the unlock period, reflecting investigative delays. Less frequent causes such as drowning, poisoning, septicaemia and maternal deaths were nearly absent during lockdown but emerged in the unlock phase. Liver-related deaths remained relatively low at 4.14% (Table 3 - see PDF). The manner of death did not significantly differ between lockdown and unlock phases (χ^2^ = 4.62, df = 4, p = 0.329). Natural deaths constituted the majority (48.82%), followed by accidental (18.10%) and suicidal deaths (13.07%), the latter rising post-lockdown. Homicidal deaths were rare (0.76%), occurring only in the unlock phase. While statistically non-significant, the data suggest post-lockdown increases in suicides and accidents could be linked to stress, mobility and societal disruption (Table 4 - see PDF). Histopathological findings also showed no significant difference between phases (χ^2^ = 6.22, df = 3, p = 0.101). "NIL" findings were most common (61.51%), with a higher frequency during unlock. Pending reports (31.61%) increased during unlock, as did preserved samples (4.84%). Histopathological abnormalities were identified in only 1.40% of cases, mostly post-lockdown (Table 5 - see PDF). Chemical analysis results similarly showed no statistically significant variation across the two phases (χ^2^ = 5.12, df = 3, p = 0.163). The majority of cases (75.14%) had no toxicological findings. Pending reports (22.12%) and preserved samples (2.29%) were largely recorded during the unlock phase. Only 0.45% of cases had confirmed absence of poisoning. These findings suggest that while laboratory testing was not significantly disrupted statistically, the operational load increased following the easing of restrictions (Table 6 - see PDF).

## Discussion:

The present study highlights the significant disruption in medicolegal autopsy patterns during the COVID-19 pandemic, especially during the lockdown phase. The drastic reduction in autopsies during lockdown (6.37%) aligns with findings from similar studies conducted in other metropolitan canters across India, where restrictions on movement, logistical barriers and reprioritization of healthcare resources led to decreased forensic casework. For instance, Kumar *et al.* reported a 54% drop in autopsies during the lockdown in Delhi [9], a trend observed in Punjab by Aggarwal *et al.* who found a 34% decline [10]. The predominance of autopsies in the 21-40 years age group in our study concurs with the demographic trends observed by Singh *et al.* who noted that this age group represents the most mobile and occupationally exposed segment of the population, thus bearing a higher risk of trauma and unnatural deaths [11]. The statistically significant age-wise shift between the lockdown and unlock phases further emphasizes how mobility and social activity correlated with exposure and mortality patterns during the pandemic [12]. Gender distribution remained consistent across phases, with males accounting for nearly 71% of the cases. This male predominance is well-documented in forensic literature, often attributed to greater exposure to outdoor activities, risky behaviours and employment in high-risk occupations. Our findings are corroborated by Kiamma and Bhatt *et al.* who reported a similar male-to-female autopsy ratio during the pandemic [13, 14]. Cause of death analysis revealed a significant rise in respiratory and cardiac causes during the unlock phase. This trend likely reflects increased healthcare-seeking behaviour post-lockdown and delayed presentation of chronic conditions. Notably, the rise in tuberculosis and pneumonia-related deaths is consistent with the findings of Semnani *et al.* who reported a resurgence of respiratory infections due to disrupted routine care and delayed diagnosis during lockdown [15]. A worrying trend was the spike in deaths by hanging during the unlock phase. Our data aligns with the study by Kar *et al.* which observed an increase in suicides during the post-lockdown period, potentially linked to economic distress, unemployment and mental health issues [16]. Similarly, trauma-related deaths surged following the resumption of vehicular and occupational activities, echoing trends reported by Patel *et al.* in Gujarat [17]. Furthermore, the high proportion of "opinion reserved/pending" cases during the unlock period points to forensic system overload and backlogs, a pattern corroborated by Mishra *et al.*, who observed delays in postmortem reporting in Odisha during the pandemic [18]. The manner of death distribution-largely unchanged between phases-still hints at a relative rise in suicides and accidents post-lockdown, a trend that, while statistically non-significant in our dataset, was echoed in studies from Maharashtra and Tamil Nadu [19, 20]. The rarity of homicidal deaths contrasts with pre-pandemic studies and suggests a decline in interpersonal violence during restricted social interaction periods [21]. Histopathological and toxicological analyses did not show significant statistical variations but revealed operational bottlenecks post-lockdown. Our observations align with findings from Van Velthuysen *et al.* who noted increased pending lab results and delays in histological assessments after the lockdown due to backlog accumulation and resource diversion [22]. This study is limited by its retrospective design and reliance on secondary data from autopsy records, which may be subject to documentation inconsistencies or missing information. As a single-center study conducted at a tertiary care hospital in a metropolitan city, the findings may not be generalizable to rural or smaller urban settings with different medicolegal caseloads and infrastructural capacities. Additionally, the high proportion of cases with pending or reserved opinions and incomplete laboratory/histopathological reports during the unlock phase limits the comprehensive interpretation of cause-specific mortality trends. The lack of direct data on COVID-19 status for the deceased also restricts correlation between SARS-CoV-2 infection and autopsy findings. In summary, our findings corroborate existing literature on the multifaceted impact of the COVID-19 pandemic on forensic pathology services. While some shifts were statistically significant, others reflect broader systemic and social disruptions.

## Conclusion:

The COVID-19 pandemic, particularly during the lockdown and subsequent unlocks phases, had a significant impact on the conduct and profile of medicolegal autopsies. A marked reduction in autopsies during lockdown reflects restricted mobility, altered healthcare priorities while post-lockdown surge in autopsies highlights the indirect health consequences of the pandemic and resumption of societal activity. These findings underscore the need for adaptive forensic protocols, robust support systems and integrated public health strategies to manage medicolegal responsibilities during large-scale health emergencies.

## Funding:

No external funding was received for this study.
